# Prognostic value of inflammation-based prognostic scores on outcome in patients undergoing continuous ambulatory peritoneal dialysis

**DOI:** 10.1186/s12882-018-1092-1

**Published:** 2018-10-26

**Authors:** Lu Cai, Jianwen Yu, Jing Yu, Yuan Peng, Habib Ullah, Chunyan Yi, Jianxiong Lin, Xiao Yang, Xueqing Yu

**Affiliations:** 1 Department of Nephrology, The First Affiliated Hospital of Sun Yat-sen University, Guangzhou, 510080 China; 2 Key Laboratory of Nephrology, Ministry of Health and Guangdong Province, Guangzhou, China; 3 Institute of Nephrology, Guangdong Medical University, Zhanjiang, China

**Keywords:** Inflammation-based prognostic scores, Continuous ambulatory peritoneal dialysis, All-cause mortality, Cardiovascular mortality

## Abstract

**Background:**

Inflammation-based prognostic scores have been used as outcome predictors in patients with cancer or on hemodialysis. However, their role in patients on continuous ambulatory peritoneal dialysis (CAPD) remains unclear. This study aimed to examine the prognostic value of inflammation-based composite scores for mortality in CAPD patients.

**Methods:**

This study was conducted in CAPD patients enrolled from January 1, 2006 to December 31, 2014 and followed until December 31, 2016. Three inflammation-based prognostic scores, including Glasgow prognostic score (GPS), prognostic nutritional index (PNI), and prognostic index (PI), were conducted in this study. The associations between these scores and all-cause or cardiovascular mortality were evaluated by Kaplan–Meier method and Cox proportional hazards models. The areas under the curve (AUC) of receiver-operating characteristic (ROC) analysis were used to determine the predictive values of mortality.

**Results:**

A total of 1501 patients were included. During a median follow-up of 38.7 (range, 21.6–62.3) months, 346 (23.1%) patients died, of which 168 (48.6%) were due to cardiovascular diseases (CVD). After adjustment for confounders, the results showed that elevated GPS, PNI, and PI scores were all independently associated with all-cause [GPS: Score 1: hazard ratio(HR) 3.94, 95% confidence interval(CI) 2.90–5.35; Score 2: HR 7.56, 95% CI 5.35–10.67; PNI: HR 1.82, 95% CI 1.36–2.43; PI: Score 1: HR 2.08, 95% CI 1.63–2.65; Score 2: HR 3.03, 95% CI 2.00–4.60)] and CVD mortality(GPS: Score 1: HR 4.41, 95% CI 2.76–7.03; Score 2: HR 9.64, 95% CI 5.72–16.26; PNI: HR 1.63, 95% CI 1.06–2.51; PI: Score 1: HR 2.57, 95% CI 1.81–3.66, Score 2: HR 3.85, 95% CI 1.99–7.46).The AUC values of GPS score were 0.798 (95% CI0.770–0.826) for all-cause mortality and 0.781 (95% CI 0.744–0.817) for CVD mortality, both of which significantly higher than those of PNI and PI scores (*P* < 0.001, respectively).

**Conclusions:**

All elevated GPS, PNI, and PI scores were independently associated with all-cause and CVD mortality. The GPS score showed better predictive value than PNI and PI scores in CAPD patients.

## Background

Peritoneal dialysis (PD) has been established as a successful treatment modality of renal replacement therapy over decades [1]. However, the mortality of PD patients remains much higher compared to general population, nearly half of which are caused by cardiovascular disease (CVD) [2, 3]. Numerous risk factors have been identified to be associated with CVD [4–7]. Among them, systemic inflammation is well recognized for its close relationship to cardiovascular morbidity and mortality [8]. We and others found that elevated C-reactive protein (CRP) levels, especially its elevated trend over time, could be independently predictive of mortality in PD population [9–11]. Importantly, inflammation drives the development of malnutrition, which may in turn amplify systemic inflammation responses, leading to a vicious cycle [12, 13]. Recently, International Society for Peritoneal Dialysis (ISPD) cardiovascular and metabolic guidelines suggest that PD patients with persistently elevated CRP should be investigated for any treatable cause of inflammation and nutritional status should be assessed within 6–8 weeks after commencement of PD for reducing the risk of CVD mortality [2]. Therefore, comprehensive assessment of inflammatory and nutritional status will help to identify patients at high risk and are crucial in the management of PD cohorts. However, standardized methods or systems available for this purpose remain to be explored.

Inflammation-based prognostic scores have been developed since last decade and successfully used to monitor patients’ status and predict outcomes in cancer management [14–20]. The Glasgow prognostic score (GPS), composed of serum CRP and albumin, has been reported as a powerful predictor for mortality in many cancer patients [14–16]. The prognostic nutritional index (PNI), which was originally developed to monitor nutritional status of perioperative patients, can predict long-term outcomes in patients with a variety of malignancy [17–19]. The prognostic index (PI), based on CRP and white blood cell (WBC) count, has also been shown to be associated with survival in advanced lung cancer patients [20]. However, few studies have investigated the association of these composite scores with outcomes in continuous ambulatory peritoneal dialysis (CAPD) patients. Therefore, the purpose of this study was to evaluate the prognostic values of these scores in CAPD patients.

## Methods

### Study participants

Patients were enrolled from PD center of The First Affiliated Hospital of Sun Yat-sen University from January 1, 2006 to December 31, 2014. Patients who had received CAPD for more than 3 months were included. Patients who were younger than 18 years old, undergone CAPD for less than 3 months, transferred from hemodialysis (HD), with a history of renal transplantation or malignancy before PD, or without data of serum CRP, albumin, or WBC count, were excluded from this study. The study was approved by the Ethics Committee of The First Affiliated Hospital of Sun Yat-sen University. All participants provided their written informed consent for this study.

### Data collection and laboratory measurements

This work was a retrospective cohort study. Baseline demographic and clinical data, including age, gender, a history of smoke, diabetes, hypertension, cardiovascular disease, were collected at the start of CAPD treatment. Diabetes and hypertension were recorded as previously defined [21]. Baseline biochemical parameters were collected 1–3 months after the initiation of PD therapy, including blood pressure (BP), hemoglobin, WBC count, serum CRP, albumin, total triglycerides, total cholesterol, low-density lipoprotein cholesterol (LDL-C), high-density lipoprotein cholesterol (HDL-C), uric acid, and creatinine. Residual renal function, in ml/min/1.73m^2^, was estimated from mean values of creatinine clearance and urea clearance and adjusted for body surface area calculated with the Gehan and George equation [6]. All measurements of biochemical parameters were performed in the biochemical laboratory of The First Affiliated Hospital of Sun Yat-sen University. The constituents of three inflammation-based prognostic scores (GPS, PNI and PI) were listed in Table 1.

**Table 1:** Inflammation-based prognostic scores

Scoring systems	Score
GPS	
CRP ≤ 10 mg/L and ALB ≥ 35 g/L	0
CRP > 10 mg/L or ALB < 35 g/L	1
CRP > 10 mg/L and ALB < 35 g/L	2
PNI	
10 × serum albumin value (g/dl) + 0.005 × peripheral lymphocyte count (/ul) ≥ 45	0
10 × serum albumin value (g/dl) + 0.005 × peripheral lymphocyte count (/ul) < 45	1
PI	
CRP ≤ 10 mg/L and WBC ≤ 11 × 10^9^/L	0
CRP ≤10 mg/L and WBC > 11 × 10^9^/L	1
CRP > 10 mg/L and WBC ≤ 11 × 10^9^/L	1
CRP > 10 mg/L and WBC > 11 × 10^9^/L	2

Abbreviations: *GPS* Glasgow Prognostic Score, *CRP* C-reactive protein, *ALB* albumin, *PNI* prognostic nutritional index, *PI* prognostic index, *WBC* white blood cell

### Outcomes

The primary endpoint of this study was all-cause mortality, and the second endpoint was CVD mortality. CVD mortality was defined as death caused by events including acute myocardial infarction, cardiac arrhythmia, congestive heart failure, atherosclerotic heart disease, cardiomyopathy, cardiac arrest, intracranial hemorrhage, cerebral infarction and peripheral vascular disease [22]. All participants were followed up until death, cessation of PD, or December 31, 2016.

### Statistical analysis

The data were presented as mean ± standard deviation for normally distributed continuous variables, median (interquartile range) for skewed continuous variables, and number (proportion) for categorical variables. The Kaplan-Meier curve was used to calculate survival rate followed by log-rank test to compare differences among groups. Univariate and multivariate Cox proportional hazards models were used to analyze the associations between prognostic scores and all-cause and CVD mortality. The multivariate Cox regression model was constructed by adjusting covariates using a backward stepwise selection procedure with a stay criterion of 0.10 (the selection cut-off value was from default in SPSS software system as well as the importance of clinical concern). Receiver-operating characteristic (ROC) analysis was performed and the area under the curve (AUC) was calculated to determine the predictive power of prognostic scores for mortality. Comparison of AUC values among groups was determined using MedCalc software version 15.0 (Broekstraat, Mariakerke, Belgium) [23]. All other statistical analyses were performed using SPSS version 22.0 for Windows (SPSS, Chicago, IL, USA). *P* < 0.05 was considered statistically significant using two-tailed tests.

## Results

### Baseline demographic and clinical characteristics

Baseline demographic and clinical characteristics of the cohort study are given in Table 2. A total of 1501 eligible CAPD patients were included in this study. The mean age was 46.4 ± 15.1 years, 59.1% were male, 21.7% had a history of diabetes mellitus. The leading cause of ESRD was primary glomerulonephritis (928, 61.8%), followed by diabetic nephropathy (292, 19.5%), hypertension (135, 9.0%) and others (146, 9.7%). The median vintage of PD was 38.7 (range, 21.6–62.3) months.

**Table 2:** Baseline characteristics of 1501 CAPD patients

Characteristics	Values
Age (years)	46.4 ± 15.1
Gender (Male)	887 (59.1%)
Smoke	253 (16.9%)
Body mass index (kg/m^2^)	21.5 ± 3.7
Systolic BP (mmHg)	136.2 ± 20.6
Diastolic BP (mmHg)	84.9 ± 14.4
Hypertension	605 (40.3%)
Diabetes mellitus	326 (21.7%)
Cardiovascular disease	249 (16.6%)
Serum albumin (g/L)	36.4 ± 5.0
Calcium (mmol/L)	2.2 ± 0.3
Phosphorus (mmol/L)	1.7 ± 0.6
iPTH (pg/mL)	289.0 (144.4–455.5)
CRP (mg/L)	1.6 (0.8–5.5)
WBC (× 10^9^/L)	6.9 ± 2.4
Lymphocyte (× 10^9^//L)	1.4 ± 0.6
Hemoglobin (g/L)	89.8 ± 22.8
Total cholesterol (mmol/L)	5.1 ± 1.4
Total triglycerides (mmol/L)	1.6 ± 1.1
HDL-C (mmol/L)	1.2 ± 0.4
LDL-C (mmol/L)	3.0 ± 1.1
Plasma uric acid (μmol/L)	430.3 ± 101.0
Plasma creatinine (μmol/L)	766.7 ± 277.5
RRF (ml/min/1.73m^2^)	3.7 ± 3.0

Abbreviations: *CAPD* continuous ambulatory peritoneal dialysis, *BP* blood pressure, *iPTH* intact parathyroid hormone, *CRP* C-reactive protein, *WBC* white blood cell, *HDL-C* high-density lipoprotein cholesterol, *LDL-C* low-density lipoprotein cholesterol, *RRF* residual renal function

During the follow-up period, 318 (21.2%) patients underwent renal transplantation, 185 (12.3%) were transferred to HD, 59 (3.9%) were transferred to other centers, 36 (2.4%) were lost to follow-up, and finally, 903 (60.2%) were followed up until the end of the study.

### Inflammation-based prognostic scores

According to the GPS scoring system, 909 (60.6%) of the 1501 patients showed a score of 0, while 456 (30.4%) and 136 (9.1%) patients had a score of 1 and 2, respectively. PNI classification revealed that 897 (59.8%) patients had a score of 1. With regard to PI, there were 253 (16.9%) and 35 (2.3%) patients who displayed a score of 1 and 2, respectively. In mortality population, larger proportions of patients were categorized into higher score groups (Table 3). Compared with non-diabetic patients, diabetic patients presented with higher scores (Table 4).

**Table 3:** Distribution of inflammation-based prognostic scores among groups

Prognostic score	All patients (*n* = 1501)	Survival patients (*n* = 1155)	All-cause mortality (*n* = 346)	CVD mortality (*n* = 168)
GPS				
0	909 (60.6%)	845 (73.2%)	64 (18.5%)	26 (15.5%)
1	456 (30.4%)	272 (23.5%)	184 (53.2%)	89 (53.0%)
2	136 (9.1%)	38 (3.3%)	98 (28.3%)	53 (31.5%)
PNI				
0	604 (40.2%)	537 (46.5%)	67 (19.4%)	29 (17.3%)
1	897 (59.8%)	618 (53.5%)	279 (80.6%)	139 (82.7%)
PI				
0	1213 (80.8%)	1016 (88.0%)	197 (56.9%)	89 (53.0%)
1	253 (16.9%)	130 (11.3%)	123 (35.6%)	68 (40.5%)
2	35 (2.3%)	9 (0.8%)	26 (7.5%)	11 (6.5%)

Abbreviations: *GPS* Glasgow Prognostic Score, *PNI* prognostic nutritional index, *PI* prognostic index, *CVD* cardiovascular disease

**Table 4:** Comparison of inflammation-based prognostic scores between diabetic and non-diabetic patients

Prognostic score	All patients (*n* = 1501)	Diabetic patients (*n* = 326)	Non-diabetic Patients (*n* = 1175)	*P* value
GPS				
0	909 (60.6%)	104 (31.9%)	805 (68.5%)	< 0.001
1	456 (30.4%)	175 (53.7%)	281 (23.9%)	
2	136 (9.1%)	47 (14.4%)	89 (7.6%)	
PNI				
0	604 (40.2%)	75 (23.0%)	529 (45.0%)	< 0.001
1	897 (59.8%)	251 (71.0%)	646 (55.0%)	
PI				
0	1213 (80.8%)	233 (71.5%)	980 (83.4%)	< 0.001
1	253 (16.9%)	79 (24.2%)	174 (14.8%)	
2	35 (2.3%)	14 (4.3%)	21 (1.8%)	

Abbreviations: *GPS* Glasgow Prognostic Score, *PNI* prognostic nutritional index, *PI* prognostic index, *CVD* cardiovascular disease

### Patient survival

**Fig. 1:**
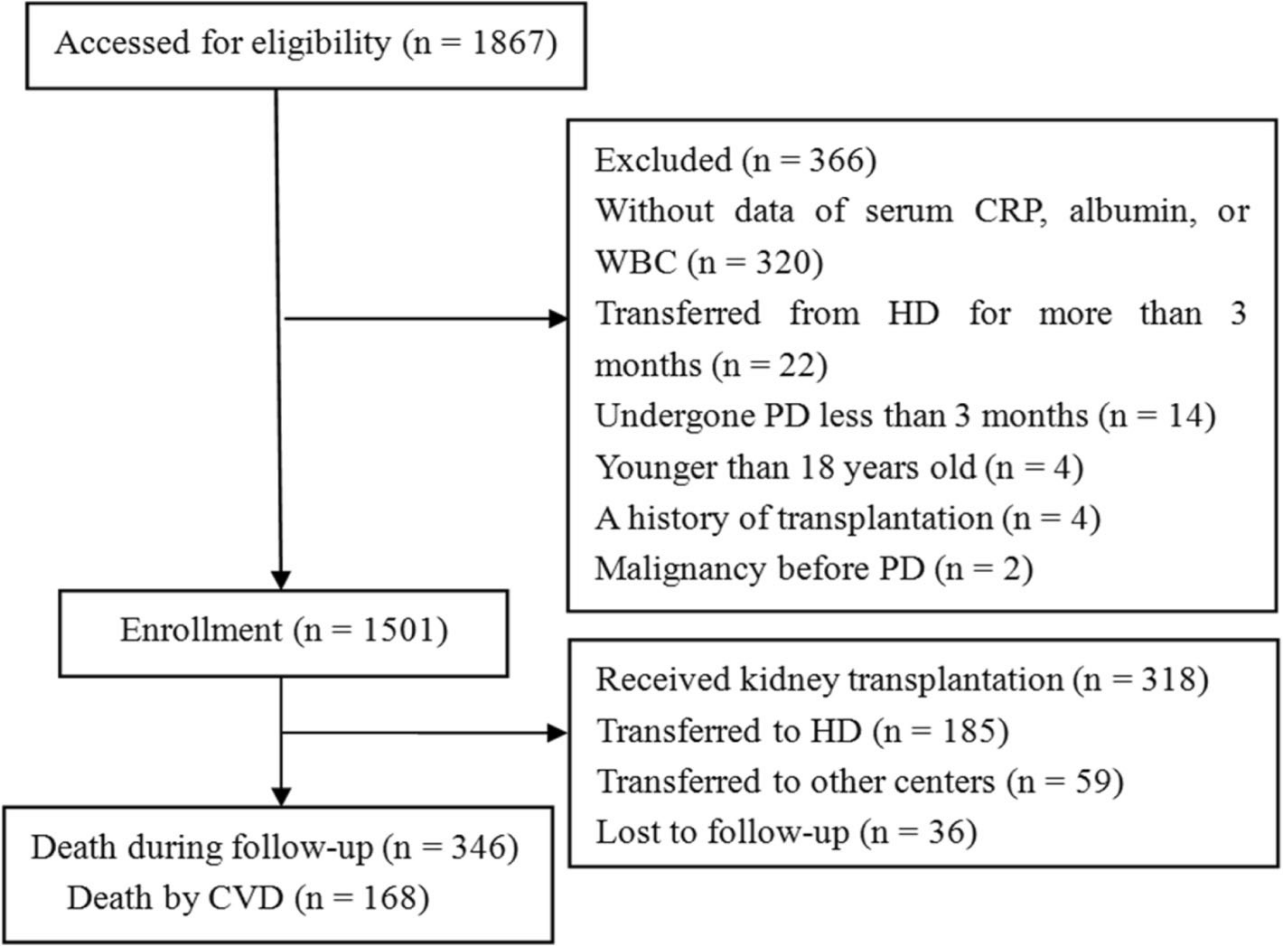
Flowchart of the patient selection process. Abbreviations: CRP, C-reactive protein; WBC, white blood cell; PD, peritoneal dialysis; HD, hemodialysis; CVD, cardiovascular disease

**Fig. 2:**
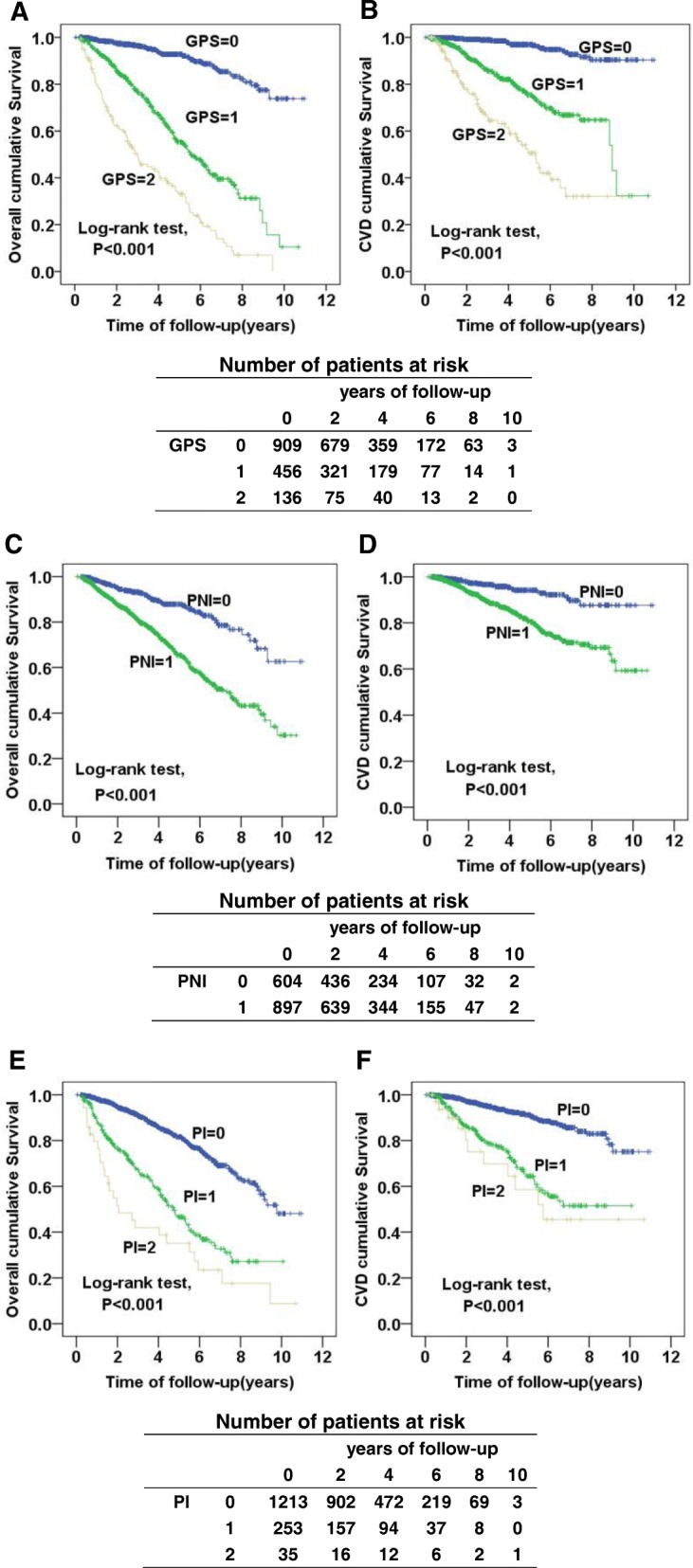
Kaplan–Meier estimates of cumulative overall (**a**, **c**, **e**) and CVD-free (**b**, **d**, **f**) survival rate according to different prognostic scores. Abbreviations: CVD, cardiovascular disease; GPS, Glasgow Prognostic Score; PNI, prognostic nutritional index; PI, prognostic index

A total of 346 deaths (23.1%) occurred, of which 168 (48.6%) were attributed to CVD (Fig. 1). Kaplan-Meier analyses indicated that the cumulative overall survival rates of patients with a GPS score of 0, 1, 2, were 93.0%, 59.6%, 27.9%, respectively (log-rank test, *P* < 0.001); the CVD survival rates were also significantly lower in patients with higher scores (score 1: 80.5%; score 2: 61.0%) than those with a score of 0 (97.1%) (log-rank test, *P* < 0.001). Elevated PNI and PI scores were also shown to be associated with reduced all-cause and CVD survival rates (Fig. 2).

Univariate cox hazards analysis revealed that increased GPS, PNI and PI scores were all significantly related to all-cause and CVD mortality (Table 5). After adjusting for covariates including age, BP, diabetes, hypertension, cardiovascular disease, infection, hemoglobin, total triglycerides, total cholesterol, LDL-C, HDL-C, uric acid, and creatinine, the patients with increased GPS scores still had a significant increased risk for overall [Score 1: hazard ratio(HR) 3.94, 95% confidence interval(CI) 2.90–5.35, *P* < 0.001; Score 2: HR 7.56, 95% CI 5.35–10.67, *P* < 0.001] and CVD mortality (Score 1: HR 4.41, 95% CI 2.76–7.03,*P* < 0.001; Score 2: HR 9.64, 95% CI 5.72–16.26, *P* < 0.001). Increased PNI and PI values were also independently predictive of all-cause and CVD mortality (Table 6).

**Table 5:** Univariate cox proportional analysis for all-cause and CVD mortality

	Univariate (All-cause mortality)		Univariate (CVD mortality)	
	HR (95%CI)	*P* value	HR (95%CI)	*P* value
Age	1.06 (1.05–1.07)	< 0.001	1.07 (1.06–1.08)	< 0.001
Gender (Male)	0.92 (0.74–1.13)	0.429	0.96 (0.71–1.30)	0.790
Smoke	1.16 (0.88–1.52)	0.300	1.18 (0.80–1.73)	0.401
Body mass index(kg/m^2^)	1.06 (1.03–1.09)	< 0.001	1.07 (1.03–1.12)	0.002
Systolic BP (mmHg)	1.01 (1.00–1.01)	0.013	1.01 (1.00–1.02)	0.003
Diastolic BP (mmHg)	0.98 (0.97–0.98)	< 0.001	0.98 (0.97–0.99)	< 0.001
Hypertension	2.86 (2.29–3.56)	< 0.001	3.92 (2.81–5.48)	< 0.001
Diabetes mellitus	3.35 (2.71–4.14)	< 0.001	4.45 (3.29–6.03)	< 0.001
Cardiovascular disease	3.54 (2.84–4.43)	< 0.001	4.96 (3.65–6.73)	< 0.001
Infection	3.46 (2.43–4.94)	< 0.001	2.64 (1.50–4.66)	0.001
Residual renal function	0.97 (0.92–1.01)	0.12	0.94 (0.88–1.01)	0.09
Calcium (mmol/L)	0.31 (0.21–0.45)	< 0.001	0.28 (0.16–0.49)	< 0.001
Phosphorus (mmol/L)	1.01 (0.85–1.20)	0.902	0.94 (0.73–1.20)	0.610
iPTH (pg/mL)	1.00 (1.00–1.00)	0.229	1.00 (1.00–1.00)	0.626
Hemoglobin (g/L)	0.99 (0.99–1.00)	0.001	0.99 (0.98–0.99)	< 0.001
Total cholesterol (mmol/L)	1.04 (0.97–1.13)	0.257	1.12 (1.02–1.23)	0.022
Total triglycerides (mmol/L)	1.12 (1.03–1.21)	0.008	1.16 (1.04–1.29)	0.007
HDL-C (mmol/L)	0.58 (0.44–0.77)	< 0.001	0.62 (0.42–0.93)	0.021
LDL-C (mmol/L)	1.05 (0.95–1.16)	0.341	1.12 (0.99–1.28)	0.070
Serum uric acid (μmol/L)	1.00 (1.00–1.00)	0.069	1.00 (1.00–1.00)	0.007
Serum creatinine (μmol/L)	1.00 (1.00–1.00)	< 0.001	1.00 (1.00–1.00)	< 0.001
GPS				
0	reference		Reference	
1	6.37 (4.79–8.48)	< 0.001	7.46 (4.81–11.56)	< 0.001
2	14.66 (10.68–20.13)	< 0.001	19.09 (11.91–30.57)	< 0.001
PNI				
0	reference		Reference	
1	2.84 (2.17–3.70)	< 0.001	3.27 (2.19–4.88)	< 0.001
PI				
0	reference		Reference	
1	3.48 (2.78–4.37)	< 0.001	4.26 (3.10–5.85)	< 0.001
2	5.29 (3.51–7.98)	< 0.001	5.08 (2.71–9.53)	< 0.001

Abbreviations: *HR* hazard ratio, *CI* confidence interval, *CAPD* continuous ambulatory peritoneal dialysis, *BP* blood pressure, *iPTH* intact parathyroid hormone, *CRP* C-reactive protein, *WBC* white blood cell, *HDL-C* high-density lipoprotein cholesterol, *LDL-C* low-density lipoprotein cholesterol, *RRF* residual renal function, *GPS* Glasgow Prognostic Score, *PNI* prognostic nutritional index, *PI* prognostic index, *CVD* cardiovascular disease

**Table 6:** Multivariate cox proportional analysis for all-cause and CVD mortality

	Multivariate (All-cause mortality)		Multivariate (CVD mortality)	
	HR (95%CI)	*P* value	HR (95%CI)	*P* value
GPS				
0	reference		Reference	
1	3.94 (2.90–5.35)	< 0.001	4.41 (2.76–7.03)	< 0.001
2	7.56 (5.35–10.67)	< 0.001	9.64 (5.72–16.26)	< 0.001
PNI				
0	Reference		reference	
1	1.82 (1.36–2.43)	< 0.001	1.63 (1.06–2.51)	0.027
PI				
0	reference		reference	
1	2.08 (1.63–2.65)	< 0.001	2.57 (1.81–3.66)	< 0.001
2	3.03 (2.00–4.60)	< 0.001	3.85 (1.99–7.46)	< 0.001

Adjustments were made for variables from the predictor variables of Table 5 using a backward stepwise cox proportional hazards model with a stay criterion of 0.10

Abbreviations: *GPS* Glasgow Prognostic Score, *PNI* prognostic nutritional index, *PI* prognostic index, *CVD* cardiovascular disease

### Comparison of prognostic values of inflammation-based scores

**Fig. 3:**
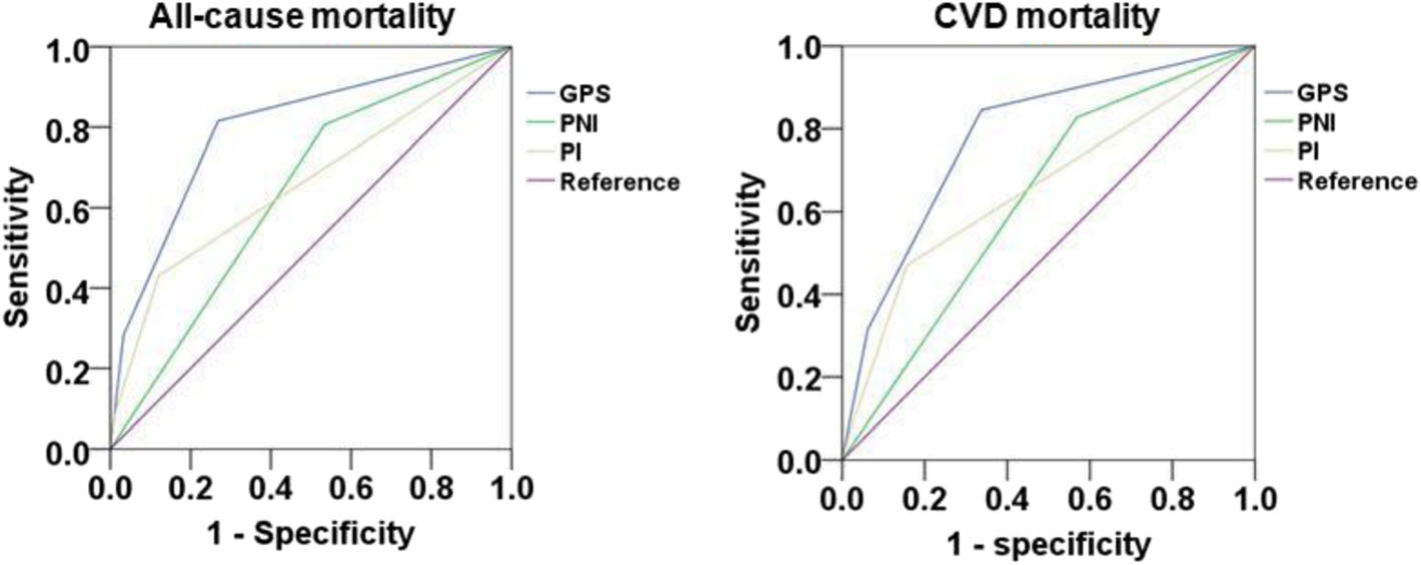
ROC curve of prognostic scores for mortality

When all-cause mortality was used as an endpoint, the area under the curve (AUC) was 0.798 (95% CI 0.770–0.826, *P* < 0.001) for GPS, 0.636 (95% CI 0.604–0.667, *P* < 0.001) for PNI, 0.658 (95% CI 0.622–0.694, *P* < 0.001) for PI. The AUC values for CVD mortality were 0.781 (95% CI 0.744–0.817, *P* < 0.001) for GPS, 0.629 (95% CI 0.589–0.670, *P* < 0.001) for PNI and 0.658 (95% CI 0.609–0.706, *P* < 0.001) for PI. By comparison of AUC values among groups, the GPS score showed a better distinguishing power for predicting all-cause and CVD mortality compared with PNI and PI (*P* < 0.001, respectively) (Fig. 3 & Table 7).

**Table 7:** Area under the ROC curve of prognostic scores for all-cause and CVD mortality

Prognostic score	Area under the ROC curve	95% CI	*P* value (vs. GPS)
All-cause mortality			
GPS	0.798	0.770–0.826	
PNI	0.636	0.604–0.667	< 0.001
PI	0.658	0.622–0.694	< 0.001
CVD mortality			
GPS	0.781	0.744–0.817	
PNI	0.629	0.589–0.670	< 0.001
PI	0.658	0.609–0.706	< 0.001

Abbreviations: *ROC* receiver-operating characteristic analysis, *HR* hazard ratio, *CI* confidence interval, *CAPD* continuous ambulatory peritoneal dialysis, *BP* blood pressure, *iPTH* intact parathyroid hormone, *CRP* C-reactive protein, *WBC* white blood cell, *HDL-C* high-density lipoprotein cholesterol, *LDL-C* low-density lipoprotein cholesterol, *RRF* residual renal function, *GPS* Glasgow Prognostic Score, *PNI* prognostic nutritional index, *PI* prognostic index, *CVD* cardiovascular disease

## Discussion

In this retrospective cohort study of 1501 CAPD patients with a median follow-up of 38.7 months, we demonstrated that increased GPS, PNI, and PI scores were all significantly related to all-cause and CVD mortality after adjustment for confounders. ROC analysis indicated that GPS had the best predictive value among these three scores system for CAPD patients.

Inflammation is prevalent in PD patients [8]. Besides acute episodes of peritonitis, micro-inflammation also constitutes an important component of systemic inflammation responses [8, 9, 12]. Micro-inflammation in PD patients may be attributed to accumulation of uremic toxins, catheter implantation, bioincompatible dialysis solution, and so on [8]. Infections in the occult areas may also play a role, such as periodontal problems [24]. Systemic inflammation status is closely related to malnutrition and atherosclerosis. These three factors interrelate with each other and form a vicious cycle, eventually leading to increased cardiovascular morbidity and mortality [9, 12, 13]. In our study, although a minor population (62/1501) had active infection during data collection period, most patients did not present obvious signs of infection. The median level of CRP of the whole cohort was in the normal range, which may support the importance of micro-inflammation.

GPS, comprising CRP and serum albumin, is a concise prognostic score that may reflect presence of both the systemic inflammatory response and deteriorating nutritional status. Inamoto et al. found the GPS was an independent prognostic factor for cancer-specific survival and overall survival after surgery with curative intent for localized upper tract urothelial carcinoma [10]. A study based on regular HD patients showed that elevated GPS was independently predictive of all-cause mortality and hospitalization during 42-month follow-up [25]. Consistent to these reports, our results showed that raised GPS values consistently related to both overall and CVD mortality in CAPD patients. The strong power for outcome prediction of this score may be attributed to the combined effects of its components. Both markers, CRP and serum albumin, have been demonstrated to be strongly associated with all-cause and CVD mortality in patients on PD [9–11, 26, 27]. However, ROC analysis revealed that GPS had the higher value than hypoalbuminemia or increased CRP alone (data not shown), which may indicate a reciprocal interaction between these two factors.

The PNI score, which is based on serum albumin and total lymphocyte count, has been developed mainly to assess the nutritional status of patients [17–19]. In this study we found that elevated PNI score was independently associated with increased risk for overall and CVD mortality in CAPD patients. To our knowledge, another 2 studies have explored the predictive effect of PNI in PD cohorts [28, 29]. One study was limited to Korean subjects and showed that the PNI score was significantly related to all-cause mortality in PD patients, which is in agreement with our result [28]. The other study reported that PNI was associated with increased risk for CVD mortality but not all-cause mortality in 345 Chinese PD patients, which is partly conflicting with our findings [29]. The discrepancy may be due to differences in sample size, definition of PNI thresholds, or confounders chosen for adjustment. The PI score is composed of CRP and WBC count and has been validated as a useful predictive factor in lung and colon cancer [20, 30]. It is also suggested that PI was related to all-cause mortality in patients on regular HD [24]. Our study added new evidence that elevated PI scores were also independently predictive of overall and CVD mortality in a large population of CAPD patients.

The prognostic values of these prognostic scores in CAPD patients were compared in our study. Results indicated that the GPS consistently exhibited a higher AUC value compared with PNI and PI scores and showed an excellent discriminatory performance for the CAPD patients. These findings were consistent with Akihiko’s report [24], in which the GPS score had the best predictive power for prognosis of HD patients. The GPS score was a combination of suitable markers for inflammation and malnutrition, while the other two were inclined to isolated aspects. These comparisons thus imply that a comprehensive monitor of both inflammatory and nutritional status may help better improve outcomes in dialysis patients. In addition, these inflammation-based prognostic scores consist of components which are routinely available with low cost.

There are some limitations in the present study. Firstly, this was a retrospective study conducted in one single center and may thus have potential selection bias. Secondly, a large number of patients without certain blood test results were excluded, making those enrolled may not be well representative for the PD population. Thirdly, we calculated the values of these scoring systems at baseline, while a time-averaged score may be better for outcome prediction. Last but not the least, a minor population of patients with active infection were included in our cohort. Although our results showed the existence of infection did not affect the prognostic significance of scoring systems, we could not exclude the possibility of other confounding effects that deranged CRP or albumin levels during infection may produce.

## Conclusions

In conclusion, the present study demonstrated that three well-standardized prognostic scores, GPS, PNI, and PI, are all independently associated with all-cause and CVD mortality in CAPD patients. In particularly, the GPS score shows the better predictive power for mortality compared to the other two scores. The GPS score may thus represent a simple and feasible tool for outcome prediction in CAPD patients.

## Abbreviations

AUC: The area under the curve
BP: Blood pressure
CAPD: Continuous ambulatory peritoneal dialysis
CI: Confidence interval
CRP: C-reactive protein
CVD: Cardiovascular disease
GPS: Glasgow Prognostic Score
HD: Hemodialysis
HDL-C: high-density lipoprotein cholesterol
HR: Hazard ratio
LDL-C: Low-density lipoprotein cholesterol
PD: Peritoneal dialysis
PI: Prognostic index
PNI: Prognostic nutritional index
ROC: Receiver-operating characteristic analysis
WBC: White blood cell
